# Musical novices perform with equal accuracy when learning to drum alone or with a peer

**DOI:** 10.1038/s41598-021-91820-0

**Published:** 2021-06-14

**Authors:** Andrea Schiavio, Jan Stupacher, Elli Xypolitaki, Richard Parncutt, Renee Timmers

**Affiliations:** 1 Centre for Systematic Musicology, University of Graz, Glacisstraße 27a, 8010 Graz, Austria; 2 Center for Music in the Brain, Department of Clinical Medicine, Aarhus University & The Royal Academy of Music Aarhus/Aalborg, Aarhus, Denmark; 3 Department of Music, The University of Sheffield, Sheffield, UK

**Keywords:** Human behaviour, Psychology

## Abstract

The capacity of expert musicians to coordinate with each other when playing in ensembles or rehearsing has been widely investigated. However, little is known about the ability of novices to achieve satisfactory coordinated behaviour when making music together. We tested whether performance accuracy differs when novices play a newly learned drumming pattern with another musically untrained individual (duo group) or alone (solo group). A comparison between musical outcomes of the two groups revealed no significant differences concerning performative accuracy. An additional, exploratory examination of the degree of mutual influence between members of the duos suggested that they reciprocally affected each other when playing together. These findings indicate that a responsive auditory feedback involving surprises introduced by human errors could be part of pedagogical settings that employ repetition or imitation, thereby facilitating coordination among novices in a less prescribed fashion.

## Introduction

When performing together, expert musicians can coordinate with each other thanks to a shared repertoire of sensorimotor skills supporting intentional attribution, communication, and prediction^1–5^. This set of capacities is thought to allow co-performers to entrain to the music’s temporal regularities^6–8^ and negotiate between individual and collective musical outcomes via different anticipatory strategies^9–11^. This can help the musicians adapt to, and manage, the vicissitudes of their partners’ playing^12^. As previous research in the field has mostly focused on skilled music performers, the mechanisms underlying the ability of joint actors who lack optimal expertise to achieve successful coordinated musical behavior (e.g., by minimizing interpersonal asynchronies), remain only partially understood.

The ability to coordinate movements to achieve a goal is arguably a universal human trait that serves a rich variety of unplanned or planned purposes at different functional levels^13,14^. Examples of spontaneous, emerging coordination can be found in situations where one person observes another individual rocking to a beat^15^, when audiences spontaneously coordinate their clapping after a show^16^, and even in contexts where agents sharing visual information in a participatory context are explicitly instructed not to coordinate^17^. Examples of planned coordinated activity, on the contrary, are often conceived of as indicative of shared intentions underlying interpersonal behavior^18^. Musical contexts can offer an ideal ground to study how joint action often involves both planned and unplanned intentions at various levels and timescales. Examples of interpersonal coordination in music range from the practice of re-producing a musical score (e.g., a duo playing a romantic repertoire recital), to more open musical dialogues centered on improvisation (e.g., a band performing free jazz). In both cases intentions may not be static or entirely pre-determined; rather, they can be, and in fact often are, constantly negotiated within the unfolding dynamics of interaction^19^. Yet they can sustain the performers’ flow and musical coherence, thus facilitating sensorimotor synchronization^20,21^.

As reported by Hove and colleagues^22^, error and variability in synchronization decreases with musical training^23^. Because performance characteristics of experts systematically differ from those of novices^24,25^, skilled musicians may exert important influences when interacting with novices. In a recent empirical study by Wolf and colleagues^26^, for example, the patterns of adaptation developed by expert musicians while playing along with novices were investigated. Their findings show that experts managed to ensure consistency of the joint performance building on their experience with the novices’ scores and performances. Put simply, coordinated behavior in expert-novice dyads was achieved because experts were able to either (i) extract temporal regularities in the novices’ playing, thereby adapting their musicking to these regularities, or (ii) anticipate challenging musical configurations so that delays or asynchronies were expected, and prompt reactions prepared. Inspired by previous research by Repp and Knoblich^27^, the authors interpret these results in terms of an offline mechanism that may reduce online cognitive load: on this view, experts are thought to mentally produce an “error matrix” where incoming sensory information is compared with the predictions generated by their internal performative model, allowing them to compensate for the novice’s suboptimal timing^26^.

In a similar vein, Schultz and Palmer^28^ examined how musical expertise shapes the ability to maintain a beat in conditions involving different auditory feedback (no feedback, hearing only oneself, only the partner, or both). By examining mixed (expert and novice) or same-level (novice and novice; expert and expert) dyads, it was found that while all pairs coherently coordinated, expert musicians’ and mixed pairs performed significantly better than dyads of non-musicians in tapping to a metronome when receiving full feedback. This suggests that auditory availability of an expert’s actions has a fundamental impact on the flexibility with which players can engage with each other, thereby improving performance accuracy. When novices performed together, on the contrary, they managed to maintain a steady beat only when using self-feedback, rather than information from the partner.

This raises the question under which conditions the capacity of novices to develop sensorimotor synchronization may be optimized. In a recent experimental study, Schiavio and colleagues^29^ demonstrated that novices learning music together can enhance their reciprocal learning ability if they practice in synchrony or in turn-taking with each other, rather than in other (e.g., imitative) learning modalities. Within such settings, social interaction—the ongoing network of self-sustaining and recursive dynamics that one is at the same time situated in and actively responsible for—“could allow an individual to overcome the limitations of their individual capacities by incorporating the complex dynamics of the interaction process into the basis of their internal activity”^30^. The present contribution continues this investigation by exploring the capacity of novices to coordinate with each other in a learning context based on joint drumming. By studying the ability to follow and maintain a stable rhythm, the complementary effects of each player on the other were examined, and their performance accuracy compared with that of participants involved in individual music-making. Assuming that collaborative musical activity in novices may not require an internal offline predictive template for error-correction (as in experts), but can rather develop under performance-facilitating contexts, we expected that joint performance would give rise to musical outcomes at least equally accurate as those produced by individual participants who synchronize with an isochronous computer-model. This would extend results reported in the previously mentioned study by Schiavio and colleagues^29^, who examined the outcomes of solo and duo learning on *individual* performances in a later test phase. In the present contribution, instead, dyads and solo learners performed either with a partner or not, enabling us to investigate both individual and collective performative settings. Gaining an understanding of how non-experts optimize coordinated behavior in these contexts can disclose novel aspects regarding our everyday capacity to take part in collaborative activity, in turn stimulating novel possibilities for musical development and learning. As such, the present study may also have important implications for research in music education, when it addresses the question of whether peer-to-peer learning could be a useful pedagogical tool for novices. If this interactive setting is as beneficial as individual training for developing musical skills in musically untrained individuals, then it can be further implemented in formal contexts, providing an opportunity for novices to interact and learn from each other. Accordingly, the present work can offer empirical support to existing pedagogical practices that push in this direction^31–33^.

## Methods

### Participants

A total of 52 adult participants were recruited to take part in the study through an announcement via the University of Graz mailing list. The sample size was comparable to a previous study using a similar design in joint and solo piano learning^29^. Four participants were excluded as they were not able to do the task correctly. This reduced the sample to 48 participants (28 women, 20 men, mean age 24.7 years, *SD* = 4.8). Thirty-two participants formed 16 pairs for the main group (*duo group*), and 16 were assigned to a control group (*solo group*). All pairs were formed by participants unknown to each other. Participants of both groups were not familiar with drumming, or music-making more generally: no one attended music lessons, regularly performed music, or had learned to play a musical instrument. They reported to listen to music on average 15.7 h per week (*SD* = 12).

### Ethics statement

The study was conducted at the Centre for Systematic Musicology of the University of Graz, Austria. All procedures were approved by the Ethical Committee at the University of Graz and were in accordance with the statements of the Declaration of Helsinki. Participants received a financial reward for their involvement in the study, and provided written informed consent.

### Stimuli and apparatus

**Figure 1:**
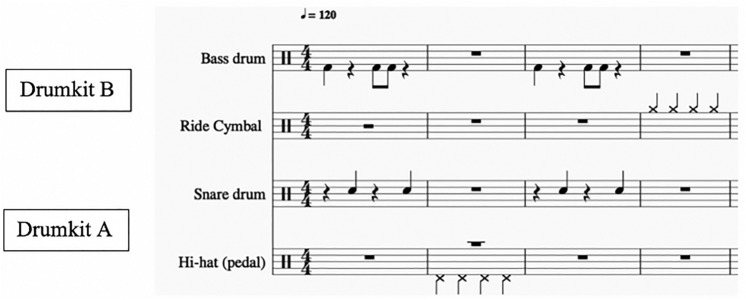
Drum pattern (stimulus 1). The score was generated with the notation software MuseScore (www.musescore.org, version 2).

**Figure 2:**
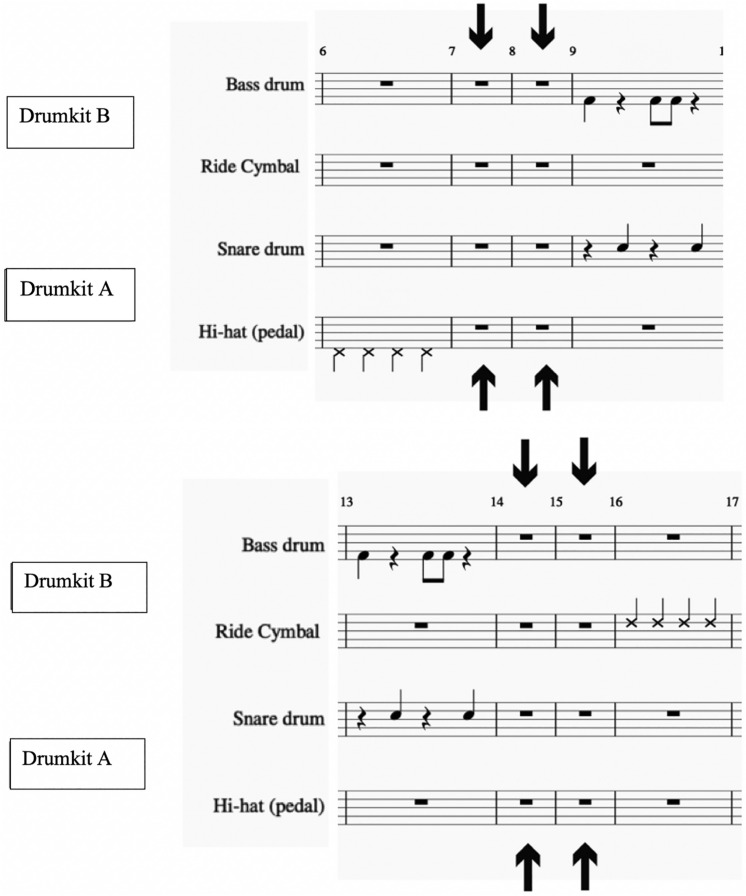
Stimulus 2: pauses at bars 7–8 and 14–15. The score was generated with the notation software MuseScore (www.musescore.org, version 2).

A simple percussion pattern incorporating independently-specifiable rhythmic levels was composed by AS for the purpose of the study (see Fig. 1). This combined 4 different drum instruments (bass drum, ride cymbal, snare drum, hi-hat), and was created with the software *MuseScore 2.* It was then extracted as .wav file and presented via headphones to participants as a continuous loop in two ways: either in its original form (stimulus 1—learning phase), or with 2-bar pauses placed every 7 bars, making the loop disrupted and unpredictable (stimulus 2—performance phase, see Fig. 2). Details about the different phases are outlined below. Two sets of E-drums (*Yamaha DTX400K*) were used for the study. Both E-drums (Drumkit A and B) were connected via USB to a laptop (*MacBook*), and to a sound interface (*Focusrite Scarlett*). The sound interface contained an internal audio mixer which helped regulate stimuli and other audio sources, sending them to different outputs as required (the researcher, participant 1 and/or participant 2). Performance data from the E-drums were recorded as MIDI using the software *Reaper64.* Before data collection, four instructional videos were recorded (using a *JVC Everion* digital camera) with a professional drummer. In an instruction video (either in German or in English), the drummer illustrated the basic technical aspects involved in playing Drumkit A, i.e., what pads to play, how to hold a stick, how to use the pedal, etc. Another instruction video was simultaneously shown to the participant sitting at Drumkit B. For the entire duration of the experiment, a researcher sat by the desk to assist participants, regulate audio settings, and ensure the procedure was carried out correctly.

### Procedure

**Figure 3:**
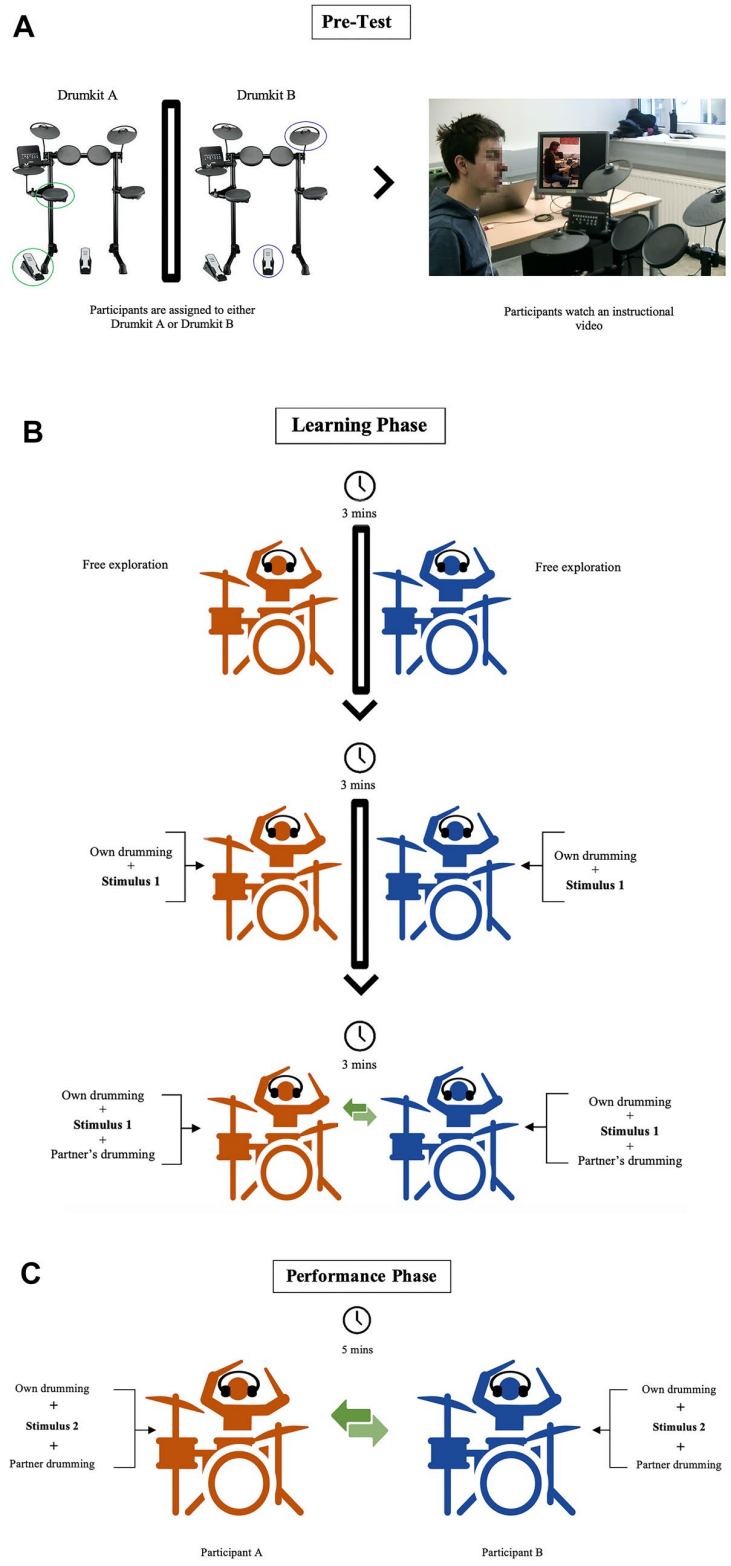
(**a**) Study design: pre-test phase*.* Participants were first assigned one of the two drumkits. They then watched a short instructional video (The picture shows a collaborator and not an actual participant of the study). (**b**) Study design: learning phase for the duo group. Participants were first instructed to freely explore the musical instrument and learn the audio-motor associations of the two pads they were asked to play for the duration of the experiment. Then, they familiarized themselves with the main drumming patterns by playing along to it both by themselves and with a partner. This figure has been designed using resources downloaded from Flaticon (www.flaticon.com, version 1). (**c**) Study design: performance phase for the duo group. Participants were asked to play with their partner for 5 min along with stimulus 2, even during the pauses. This figure has been designed using resources downloaded from Flaticon (www.flaticon.com, version 1).

The experiment took place in a dedicated room at the Centre for Systematic Musicology of the University of Graz, Austria. The design of the study is depicted in Fig. 3a–c. This involved a pre-test phase, followed by a learning phase, and a performance phase. At the end of the test, all participants were asked to complete a questionnaire.

#### Pre-test

**Figure 4:**
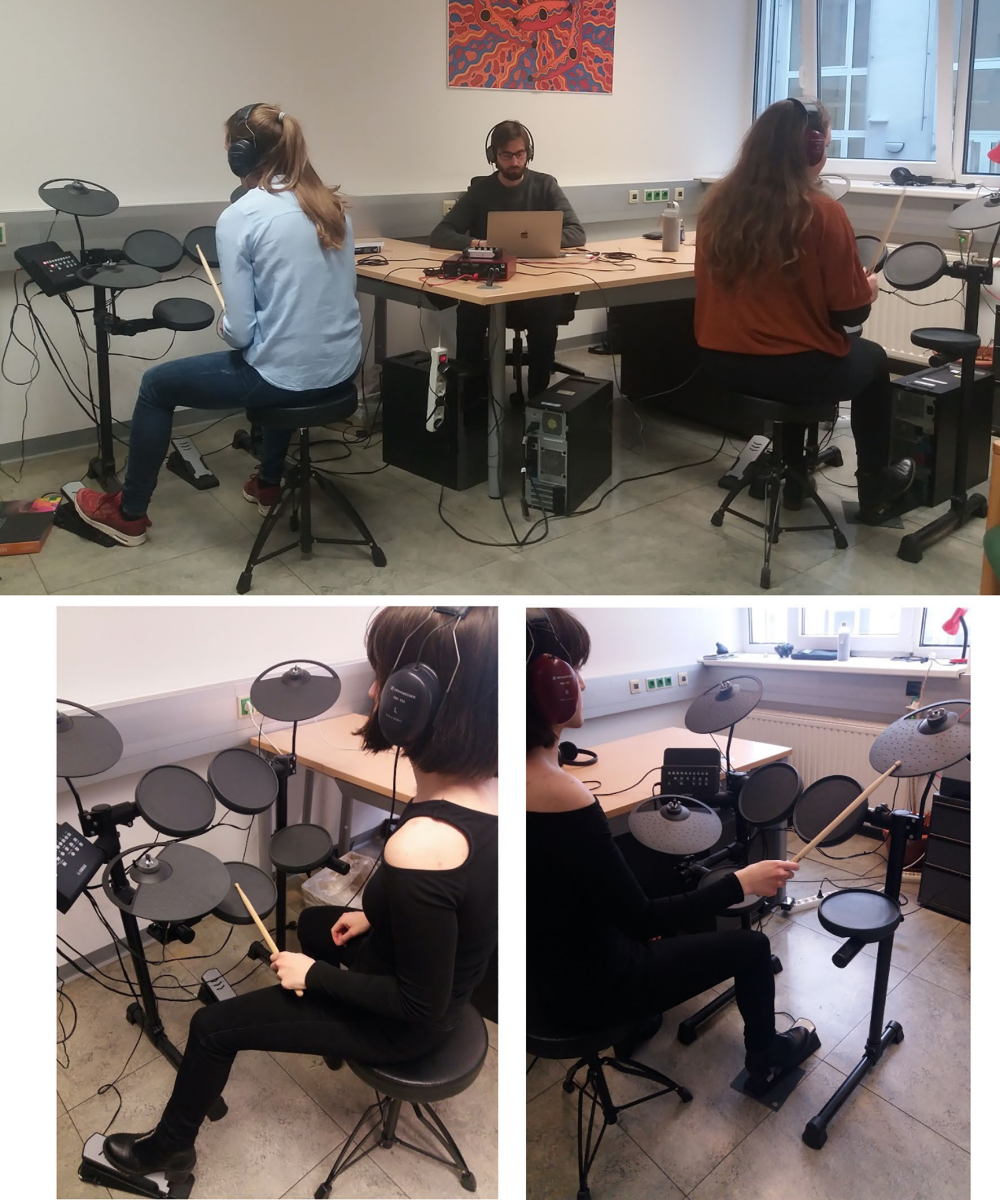
Experimental setting. Participants playing Drumkit A (on the left) only used their left hand and left foot to play snare drum and hi-hat pedal. Conversely, those playing Drumkit B (on the right) only used their right hand (to play ride cymbal) and right foot (bass drum). Participants could not see each other as they were giving their back to their partners. Participants in the control group did all tasks by themselves. They were asked to play either Drumkit A or Drumkit B, using the same pads assigned for participants in the duo group. (The picture above depicts author 1 at the center with two participants photographed from behind, so that their face will not be visible. In the picture below, author 3 illustrates in more details how the task was done).

After being instructed by the experimenter about the aims of the study, and having signed the consent form, participants from the duo group were asked to choose one of the two drumkits and, as mutual agreement was reached, they sat behind it. Participants of the solo group were assigned Drumkit A or Drumkit B by the experimenter. Sitting at the drums, all participants individually watched a 1-min instructional video that introduced them to the different pads of the E-drum with relevant examples. The video allowed the participant to associate sounds and actions to their respective pads. Two pads were assigned to each participant, depending on which E-drum they sat at: *hi-hat pedal* and *snare drum* were the pads for Drumkit A, to be played with left foot and left hand; *ride cymbal* and *bass drum* were the pads for Drumkit B, to be played with right hand and right foot. Pairs of participants (n = 32) sat at the drums with backs at each other (see Fig. 4). Participants from the solo group (n = 16) took part in the study individually, with half of them playing Drumkit A and half Drumkit B.

#### Learning phase

The learning phase comprised three different parts: (i) at the beginning, all participants were given three minutes to gain familiarity with their assigned sounds/pads associations by freely exploring the E-drums. During this explorative phase, participants were only able to listen to their own sounds; (ii) the drumming feedback in its original form (*stimulus 1*) was then presented to the participants for three minutes. Participants were asked to recognize the sounds of their own pads in the auditory stimulus and play along with them (i.e., to play their pads in synch with the stimulus, ignoring the sounds of the other two instruments, which were assigned to the other participant). Again, participants were not able to hear their partners at this stage; (iii) in the last part of the learning phase, which also lasted for three minutes and also involved *stimulus 1*, participants of the duo group were able to hear themselves as well as their partners playing. Participants of the solo group, instead, repeated part ii.

#### Performance phase

In the performance phase, an altered version of the stimulus was used (*stimulus 2*), and participants were asked to play along it as accurately as possible. Stimulus 2 involved the exact same loop of the learning phase, this time presented for a duration of five minutes (150 bars). However, 2-bar pauses (lasting for 4 s) were systematically included in the stimulus, hereafter called *break segments*. Each break segment was placed every seven bars (i.e., starting at bars 7, 14, 21, 28…147) giving rise to a total of 21, 4-s break segments, in which participants did not hear the stimulus. Because the periodicity of this manipulation was seven measures (against the four of the loop), each pause started with an articulation different from the previous one (see again Fig. 2). For example, the first break segment (bars 7–8) begins when the bass drum is supposed to be heard; the second break segment (bars 14–15) with the hi-hat; the third break segment (bars 21–22) again with the bass drum, and the fourth break segment (bars 28–29) with the ride cymbal. Participants were asked to play along with the stimulus—and keep playing during the break segments—for five minutes, always being able to hear each other as in the learning phase (iii). Similarly, participants of the solo group were asked to keep playing their own parts along with the stimulus and during the break segments. For the analysis of the performances, the break segments were treated as if the sound would have continued, i.e., performed notes were compared against the pattern that would have followed in the break segment (see Data Analysis section for more detail).

#### Post-test

Having completed the experiment, a questionnaire containing sets of Likert-scale items (ranging from 1 to 7), was administered to all participants. As reported in more detail below, the questionnaire included items such as nervousness as well as quality and satisfaction with their own learning. By asking participants from both groups to respond to these questions, we were able to compare a range of different qualitative aspects of their learning and performative experience.

### Data analysis

#### Comparison of solo versus duo performances

To assess the quality of drumming performances, three dependent variables were computed: (i) mean absolute asynchrony between performance and stimulus, indicating how tight participants were playing in relation to the stimulus; (ii) standard deviation (SD) of asynchronies between performance and stimulus, indicating how stable participants were playing with the stimulus; and (iii) the similarity between notes played in the performance and the stimulus, indicating how well participants followed the pattern of individual instruments on the drumkit. For all of these measures, double hits, defined as note onsets following another note onset in a time window shorter than 125 ms (i.e., shorter than a sixteenth note), were removed. The whole performances were divided into two groups of segments differentiating between play-along and break segments (see Fig. 2). This process gave rise to 21 play-along segments (the last play-along segment was skipped) and 21 break segments. For the computation of absolute mean asynchronies and SD of asynchronies, onsets in the performance closest to the stimulus were selected. If this interval was longer than 500 ms (i.e., longer than a quarter note) that asynchrony value was removed from the analysis (3.4% in play-along segments and 5.7% in break segments). This cleaning step assured that onsets that were missed completely did not affect the asynchrony analyses. In the break segments, absolute asynchronies were computed in relation to a “hypothetical” correct performance, that is, as if the stimulus did not involve pauses. Similarity between notes played during performance and the stimulus was calculated with the *edr* (edit distance on real signals) function in MATLAB (MathWorks, Natick, MA). This function returned the number of onsets that a participant played on the wrong part of the drumkit, missed, or added a hit in comparison to the stimulus. For the computation of *edr*, all data were used without applying the data cleaning steps used for the asynchrony measures. In the break segments, *edr* was calculated in relation to a “hypothetical” correct performance, that is, as if the stimulus did not involve pauses. To account for different lengths of the segments, the edit distance outcomes were divided by the total number of notes in the original version of the corresponding segment. For each of the three dependent variables (mean absolute asynchrony, SD of asynchronies, and edit distance) a 2 × 2 ANOVA with the within-subjects effect of *play-along vs. break* and the between-subjects effect of *solo versus duo performance* was computed with the software R (www.r-project.org) with one mean value over all segments per participant.

#### Exploratory analysis: reciprocal influence in duo performances

In an exploratory analysis, mean asynchronies with the stimulus for every participant in the duo performances were computed for the 21 play-along segments and the 21 break segments. These mean asynchrony values indicate if a participant was playing mostly ahead (negative mean asynchrony) or mostly behind (positive mean asynchrony) compared to the original stimulus in a segment. To have an indication whether participants might have influenced each other’s timing, we computed Pearson’s correlations between the mean asynchronies of the participant using Drumkit A and the participant using Drumkit B in a duo. This was done separately for play-along and break segments. As a control, each Drumkit A participant’s mean asynchronies were correlated with the mean asynchronies from the participant playing Drumkit B in the duo that was tested in the following data collection session (e.g., Duo 2, Drumkit A and Duo 3, Drumkit B). One-sample t-tests were used to compare the resulting correlation coefficients for the “real pairs” (*ensemble partners*) and “stranger pairs” (*randomly selected participants*) against zero. Values significantly greater than zero indicate that, overall, the asynchronies of the performances were associated, that is, that partners influenced each other.

## Results

### Comparison of solo versus duo performances

**Figure 5:**
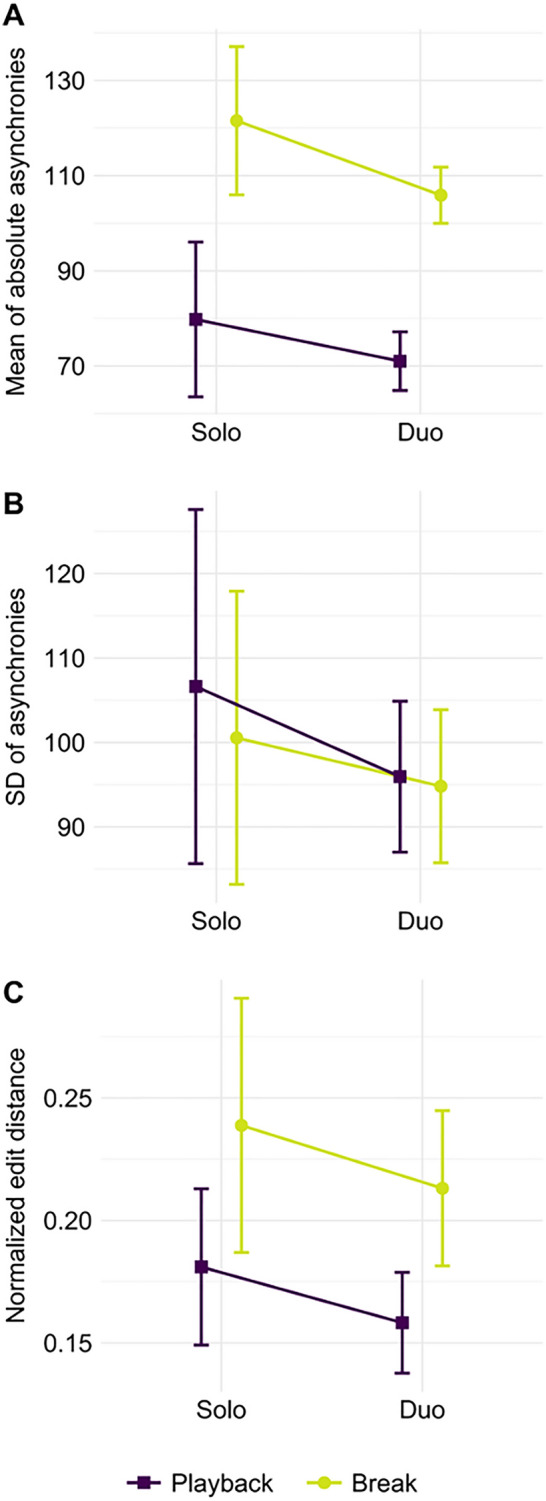
Measures of performance quality in duo and solo performances. (**a**) Mean of absolute asynchronies. (**b**) Mean of standard deviations of asynchronies. (**c**) Mean of normalized edit distances indicating how many times the wrong pads were played while drumming. Error bars represent + / − 1 SE.

Absolute asynchronies (Fig. 5a), indicating how tight participants were playing with the original stimulus, were larger in break segments than in play-along segments (*F*(1,46) = 85.42, *p* < 0.001, *η*^2^ = 0.65). Absolute asynchronies did not significantly differ between solo and duo performances (*F*(1,46) = 0.83, *p* = 0.368, *η*^2^ = 0.02). The two factors did not show a significant interaction (*F*(1,46) = 0.69, *p* = 0.410, *η*^2^ = 0.01). The standard deviation of asynchronies (Fig. 5b) showed no significant main effects and no interaction (all F < 0.7, all *p* > 0.4). Normalized edit distances (Fig. 5c), indicating how many incorrect notes participants were playing, were larger in break segments than in play-along segments (*F*(1,46) = 11.76, *p* = 0.001, *η*^2^ = 0.20). Normalized edit distances did not significantly differ between solo and duo performances (*F*(1,46) = 0.28, *p* = 0.598, *η*^2^ = 0.01). The two factors did not show a significant interaction (*F*(1,46) = 0.01, *p* = 0.930, *η*^2^ < 0.01). A similar analysis without data cleaning supported these results. Only one statistical outcome differed from the main analysis, making the findings even stronger: without data cleaning, absolute asynchronies were significantly larger in solo compared to duo performances (see Supplementary Information).

### Exploratory analysis: reciprocal influence in duo performances

**Figure 6:**
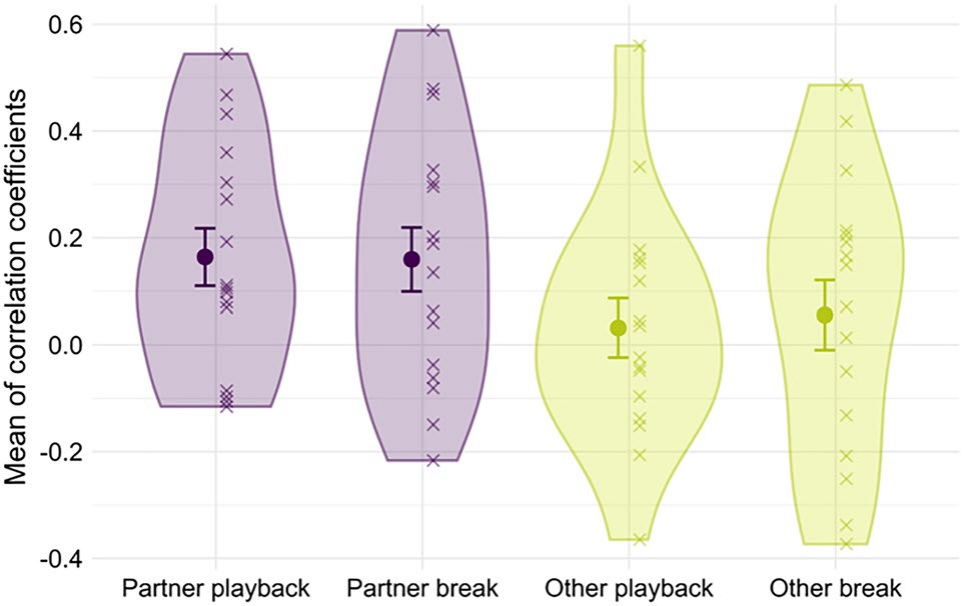
Exploratory analysis of the mutual influence of performers*.* Mean (•) and individual ( ×) values of Pearson’s correlation coefficients between the mean asynchronies of “real pairs” (ensemble partner) and “stranger pairs” (participants from different duos matched as control condition) during play-along and breaks. Values higher than zero suggest reciprocal influence. The violin plots show the probability density of the data. Error bars represent + / − 1 *SE*.

T-tests against zero indicate that the mean asynchronies in each segment of the two partners in a duo were positively correlated in play-along (*t*(15) = 3.06, *p* = 0.008, *d* = 0.77) and break segments (*t*(15) = 2.67, *p* = 0.018, *d* = 0.67) with mean correlation coefficients of *r* = 0.16 in both cases. In random pairings with other participants, correlation coefficients did not significantly differ from zero (both *t* < 0.9, both *p* > 0.4) with mean correlation coefficients of *r* < 0.06. Figure 6 offers an overview of the results and the data of the individual pairs. A similar analysis without data cleaning supported these results (see Supplementary Information). During play-along segments, the comparison of correlation coefficients with a partner versus a stranger revealed a distinct but nonsignificant difference (mean *r* = 0.16 vs. mean *r* = 0.03; *t*(30) = 1.71, *p* = 0.096). Break segments showed a similarly high but also nonsignificant difference (mean *r* = 0.16 with partner vs. mean *r* = 0.06 with stranger; *t*(30) = 1.17, *p* = 0.251).

### Questionnaires

After the performance phase, all participants were asked to complete a post-test questionnaire based on sets of Likert-scale items (ranging from 1 to 7). The instrument was designed to assess various aspects of their drumming experience during the experiment. Participants from both duo and solo groups generally enjoyed learning a new drumming pattern, with an average score of 6.0 (*SD* = 1.4) for the former and 6.5 for the latter (*SD* = 0.6). Satisfaction with their own learning was ranked M = 4.7 (*SD* = 1.4) for participants of the duo group, whereas it was *M* = 3.9 (*SD* = 1.6) for participants of the solo group. Members of the duo group were on average satisfied with the learning ability of their partners, ranking them 5.6 (*SD* = 0.8). They also ranked the overall quality of their moment-to-moment interaction during the performance task as 5.6 (*SD* = 1.0). Nervousness during the performance phase was 3.6 (*SD* = 1.8) for the duo group and 3.2 (*SD* = 1.7) for the solo group. Finally, participants were asked to indicate how much this experience motivated them to learn music in the future. The average of the duo group was 5.7 (*SD* = 1.0); the average of the solo group was 6.2 (*SD* = 0.9). Individual Wilcoxon rank sum tests indicated that none of the ratings differed significantly between the duo and solo group (all *p* > 0.07). Spearman's rank correlations indicated no significant association between the performance measures absolute asynchronies and standard deviation of asynchronies and the ratings (all *p* > 0.08).

## Discussion and conclusion

The present study examined whether performance accuracy differs when novices play a newly learned drumming pattern with another musically untrained individual or by themselves. Through an exploratory analysis, we have additionally examined the degree of mutual influence between musically untrained individuals.

We assessed the accuracy of each performance in terms of asynchronies between the target stimulus and the participants’ musical outcomes, and compared dyads (duo group) with individual learners (solo group). We found that drumming along with the stimulus (i.e., during play-along segments) resulted in smaller asynchronies and more correct notes than drumming when the stimulus was not heard (i.e., during silent breaks), even if the drumming actions were the exact same. This effect was observed in both duo and solo groups. Performances of both groups also did not significantly differ when looking at the accuracy by which the pads of the E-drums were played, that is, the ability to play the correct pad (e.g., the snare drum) at the right time, as opposed to a different pad (e.g., the hi-hat pedal). Further analyzing duo performances, our results suggest that when one participant was playing ahead of the target stimulus (i.e., anticipating the hit), the other partner was ahead too. Similarly, when one participant was behind the target stimulus (i.e., playing with delay), the other partner was behind too. Taken together, these findings provide rich insights into the dynamics of participatory music-making and learning for musically untrained participants. The present contribution resonates with existing research that highlights the key role of the external beat in optimizing performance accuracy^28^; that postulates an important similarity between musical outcomes and experiences developed across individual and collective learning settings^29,34^; and that focuses on the unfolding patterns of reciprocal adaptation that take place during joint-performance in music and beyond^35–37^. Being more accurate when drumming along with the stimulus compared to the silent breaks suggests that the capacity of novices to generate the necessary sequences of drumming actions is enhanced when engaging with relevant external auditory cues. These auditory cues could be thus thought to optimize the action-perception couplings of participants who lack relevant musical expertise, offering them valuable support to enable fluidity and accuracy of their performance. As we see next, the tendency to rely on an external stimulus plays a key role in driving coordinated behavior. Conceptually, this may help us trade the focus on internalized models (which novices may not yet build with adequate precision) for a more *extended* perspective in which the ability to follow and sustain a target stimulus is decentralized and distributed across internal and external resources^38–40^.

The lack of significant differences in performance accuracy between dyads and solo learners aligns with the previously mentioned work by Schiavio and colleagues^29^. In their study, it was found that learning new melodies on a piano together with another novice would give rise to musical outcomes exhibiting the same level of accuracy as those produced by participants who learned the same melodies individually. Such findings are particularly interesting for music education research, as peer-to-peer learning contexts have been increasingly explored in recent years^41,42^. While individual tuitions with music teachers have been long considered as the most efficient pedagogical setting (at least in Western classical contexts), recent contributions have highlighted the positive impact other novices can exert on each other, suggesting that musical skill acquisition can in fact benefit from more open learning environments in which students can participate in each other’s learning^43,44^. It should be noted that such insight is not meant to downplay the efficiency of longstanding musical learning traditions, which indeed have been proven solid in many aspects. Rather, it is offered to stimulate the implementation of complementary pedagogical strategies where learners, from their very first musical steps, can also work together and learn from each other^31,32^.

That said, it should be noted that participants who formed the dyads were unknown to each other. As such, it remains unclear whether our findings would be replicated with pairs who knew each other well. The idea that individual and collective forms of learning may give rise to similarly accurate musical outcomes and experiences is further confirmed by the analysis of the questionnaire administered in the present study. Again, no significant difference was found between groups, suggesting that individual forms of musical learning are not the only valid contexts in which students can flourish and develop their musical skills. This aligns with a recent qualitative study where verbal descriptions of music students concerning instrumental technique, expressivity, and communication were examined^34^. In the latter, it was found that although individual and participatory forms of musical learning certainly have important organizational differences, they also present consistent overlaps. Indeed, the sample of students interviewed in this study reported that in both contexts one cannot easily isolate instrumental technique from communication or expressivity, pointing again to a structural similarity between these settings—an insight also confirmed by the quantitative measures of accuracy of the present experiment.

The capacity of novices to influence each other is supported by an exploratory analysis, which might help capture the flexible nature of coordinated behavior at the core of participatory music-making. In a correlation analysis, we observed how dyads were prone to follow each other while performing—even thought they were explicitly instructed to play along with the target stimulus as accurately as possible. On the one hand, it is perhaps not surprising that novices tended to compensate for the absence of an external sensory reference by relying on the partner during the break segments; on the other hand, members of the dyads appeared to mutually influence each other (departing from the target stimulus), also during play-along times. A potential explanation might be that musically untrained individuals may display a natural inclination to follow a beat—in this case, that produced by the partner—which perhaps feels more “natural” and “responsive” than the target stimulus we presented, even if their sound qualities were hardly distinguishable. Moreover, our participants were not able to see each other when learning and performing together. The absence of visual references and the repetitive nature of the auditory stimulus, it might be suggested, could have made participants more sensitive to subtle changes in the auditory signal: as soon as a delay or anticipation was detected in the partner’s playing, a complementary drumming action was produced. Notably, these adaptations were not systematically initiated by one member of the dyad, but rather reflect a more continuous process of small-scale negotiation where both participants were consistently following one another. This partially resonates with existing work by Konvalinka and colleagues^12^, in which it was found that coordinated behavior in joint tapping was equally good when achieved with a partner that is “unpredictable but responsive” as well as with an external repetitive beat (“predictable but nonresponsive”), but was worst when participants were forced into a leader–follower scenario. This involved a form of “unidirectional” coordination, in which the degree of auditory coupling was manipulated so that participants were only able to hear the beats generated by their experimental partner. The lack of emerging leader–follower patterns in such contexts opens up possibilities to rethink teaching strategies based on repetition and imitation of the teacher, encouraging instead the development of approaches to musical skill acquisition based on novelty seeking, exploration, collaboration, and creativity^33,45–48^. Future research should expand the current exploratory approach to test whether the findings of reciprocal influence are replicable.

In sum, the present experiment yields promising results that have implications for musical skill acquisition, suggesting that learning music with other peers can be as beneficial as more traditional settings based on individual tuition. Learning music together can be a valuable resource to enrich one’s musical skills, and provides an excellent research tool to explore our capacity to share experiences and develop coordinated behavior across a variety of settings. As the ability to correctly synchronize with a beat may take years to develop^49^, it is of crucial importance to examine within ecologically valid settings how it emerges and flourishes in musically rich contexts. We thus hope to inspire future research to look further at this phenomenon and its early manifestations.

## Supplementary Information

Supplementary Information.

## Data Availability

The data supporting the findings of these studies are available from the corresponding author upon request.

## References

[1] Loehr JD, Palmer C (2011). Temporal coordination between performing musicians. Q. J. Exp. Psychol..

[2] Maes P-J (2016). Sensorimotor grounding of musical embodiment and the role of prediction: a review. Front. Psychol..

[3] Novembre G, Keller PE (2011). A grammar of action generates predictions in skilled musicians. Conscious. Cognit. Int. J..

[4] Phillips-Silver J, Trainor LJ (2007). Hearing what the body feels: auditory encoding of rhythmic movement. Cognition.

[5] Timmers R, MacRitchie J, Schabrun SM, Thapa T, Varlet M, Keller PE (2020). Neural multimodal integration underlying synchronization with a co-performer in music: influences of motor expertise and visual information. Neurosci. Lett..

[6] Large E (2000). On synchronizing movements to music. Hum. Mov. Sci..

[7] Large E, Grondin S (2008). Resonating to musical rhythm: theory and experiment. Psychology of Time.

[8] London J (2012). Hearing in Time: Psychological Aspects of Musical Meter.

[9] Bishop L, Bailes F, Dean RT (2013). Musical imagery and the planning of dynamics and articulation during performance. Music Percept..

[10] Bishop L, Bailes F, Dean RT (2014). Performing musical dynamics: How crucial are musical imagery and auditory feedback for expert and novice musicians?. Music Percept..

[11] Loehr JD, Kourtis D, Vesper C, Sebanz N, Knoblich G (2013). Monitoring individual and joint action outcomes in duet music performance. J. Cogn. Neurosci..

[12] Konvalinka I, Vuust P, Roepstorff A, Frith CD (2010). Follow you, follow me: continuous mutual prediction and adaptation in joint tapping. Q. J. Exp. Psychol..

[13] Butterfill S, Jankovic M, Ludwig K (2018). Coordinating joint action. Routledge Handbooks in Philosophy. The Routledge Handbook of Collective Intentionality.

[14] Riley MA, Richardson MJ, Shockley K, Ramenzoni VC (2011). Interpersonal synergies. Front. Psychol..

[15] Demos AP, Chaffin R, Begosh KT, Daniels JR, Marsh KL (2012). Rocking to the beat: effects of music and partner’s movements on spontaneous interpersonal coordination. J. Exp. Psychol. Gen..

[16] Néda Z, Ravasz E, Brechet Y, Vicsek T, Barabási A-L (2000). Self-organizing processes: the sound of many hands clapping. Nature.

[17] Issartel J, Marin L, Cadopi M (2007). Unintended interpersonal coordination: “Can we march to the beat of our own drum?”. Neurosci. Lett..

[18] Gilbert M (2009). Shared intention and personal intentions. Philos. Stud..

[19] Schiavio A, Høffding S (2015). Playing together without communicating? A pre-reflective and enactive account of joint musical performance. Music. Sci..

[20] D'Ausilio A, Novembre G, Fadiga L, Keller PE (2015). What can music tell us about social interaction?. Trends Cogn. Sci..

[21] Keller PE, Novembre G, Hove MJ (2014). Rhythm in joint action: psychological and neurophysiological mechanisms for real-time interpersonal coordination. Philos. Trans. R. Soc. Lond. Ser. B Biol. Sci..

[22] Hove MJ, Keller PE, Krumhansl CL (2007). Sensorimotor synchronization with chords containing tone-onset asynchronies. Percept. Psychophys..

[23] Repp BH, Penel A (2002). Auditory dominance in temporal processing: new evidence from synchronization with simultaneous visual and auditory sequences. J. Exp. Psychol. Hum. Percept. Perform..

[24] Aoki T, Furuya S, Kinoshita H (2005). Finger-tapping ability in male and female pianists and nonmusician controls. Mot. Control.

[25] Loehr JD, Palmer C (2007). Cognitive and biomechanical influences in pianists’ finger tapping. Exp. Brain Res..

[26] Wolf T, Sebanz N, Knoblich G (2018). Joint action coordination in expert-novice pairs: Can experts predict novices’ suboptimal timing?. Cognition.

[27] Repp BH, Knoblich G (2004). Perceiving action identity: how pianists recognize their own performances. Psychol. Sci..

[28] Schultz BG, Palmer C (2019). The roles of musical expertise and sensory feedback in beat keeping and joint action. Psychol. Res..

[29] Schiavio A, Stupacher J, Parncutt R, Timmers R (2020). Learning music from each other. Synchronization, turn-taking, or imitation?. Music Percept..

[30] Candadai M, Setzler M, Izquierdo EJ, Froese T (2019). Embodied dyadic interaction increases complexity of neural dynamics: a minimal agent-based simulation model. Front. Psychol..

[31] Haddon L, Burnard P (2017). Creative Teaching for Creative Learning in Higher Academic Music Education.

[32] Hanken IM (2016). Peer learning in specialist higher music education. Arts Hum. High. Educ..

[33] Borgo D (2007). Free jazz in the classroom: an ecological approach to music education. Jazz Perspect..

[34] Schiavio A, van der Schyff D, Biasutti M, Moran N, Parncutt R (2019). Instrumental technique, expressivity, and communication. A qualitative study on learning music in individual and collective settings. Front. Psychol..

[35] Knoblich G, Jordan JS (2003). Action coordination in groups and individuals: learning anticipatory control. J. Exp. Psychol. Learn. Mem. Cogn..

[36] Kourtis D, Sebanz N, Knoblich G (2013). Predictive representation of other people's actions in joint action planning: an EEG study. Soc. Neurosci..

[37] Vuust P, Witek MAG (2014). Rhythmic complexity and predictive coding: a novel approach to modeling rhythm and meter perception in music. Front. Psychol..

[38] Krueger J (2011). Extended cognition and the space of social interaction. Conscious. Cogn..

[39] Krueger J (2014). Affordances and the musically extended mind. Front. Psychol..

[40] Ryan K, Schiavio A (2019). Extended musicking, extended mind, extended agency. Notes on the third wave. New Ideas Psychol..

[41] Nielsen SG, Johansen GG, Jørgensen H (2018). Peer learning in instrumental practicing. Front. Psychol..

[42] Overy K (2012). Making music in a group: synchronization and shared experience. Ann. N. Y. Acad. Sci..

[43] Gaunt H, Westerlund H (2013). Collaborative Learning in Higher Music Education.

[44] Schiavio A, van der Schyff D, Gande A, Kruse-Weber S (2019). Negotiating individuality and collectivity in community music. A qualitative case study. Psychol. Music.

[45] Bowman W, Bresler L (2004). Cognition and the body: perspectives from music education. Knowing Bodies, Moving Minds: Toward Embodied Teaching and Learning.

[46] Peñalba A, Martinez L, Schiavio A (2020). The Active Musical Room. Fostering sensorimotor discoveries and musical creativity in toddlers. J. Res. Music Educ..

[47] van der Schyff D, Schiavio A, Walton A, Velardo V, Chemero T (2018). Musical creativity and the embodied mind. Exploring the possibilities of 4E cognition and dynamical systems theory. Music Sci..

[48] Timmers R, Sadakata M, Desain P (2012). The role of visual feedback and creative exploration for the improvement of timing accuracy in performing musical ornaments. Music Percept..

[49] McAuley JD, Jones MR, Holub S, Johnston HM, Miller NS (2006). The time of our lives: lifespan development of timing and event tracking. J. Exp. Psychol. Gen..

