# Supplementary material for: Musical novices perform with equal accuracy when learning to drum alone or with a peer
Source: Sci Rep. 2021 Jun 14;11:12422. doi: 10.1038/s41598-021-91820-0 (PMC8203685; doi:10.1038/s41598-021-91820-0)
Supplement: Supplementary file 1 — Supplementary Information. [file 41598_2021_91820_MOESM1_ESM.docx]

**Supplementary Information**

**Musical novices perform with equal accuracy when learning to drum alone or with a peer**

Andrea Schiavio ^[[1]](#footnote-1)^, Jan Stupacher ^[[2]](#footnote-2)^, Elli Xypolitaki ^[[3]](#footnote-3)^, Richard Parncutt ^1^, & Renee Timmers ^3^

**Corresponding author**

Andrea Schiavio
Centre for Systematic Musicology, University of Graz

Glacissstraße 27a, 8010 Graz, Austria

andrea.schiavio@uni-graz.at

**Comparison of Solo vs. Duo Performances without Data Cleaning**

Two dependent variables were used to assess the timing of drumming performances: mean absolute asynchrony between performance and stimulus, indicating how tight participants were playing in relation to the stimulus, and standard deviation (SD) of asynchronies between performance and stimulus, indicating how stable participants were playing with the stimulus. Similar to the analysis reported in the main text, double hits, defined as note onsets following another note onset in a time window shorter than 125 ms (i.e., shorter than a sixteenth note), were removed. For the computation of absolute mean asynchronies and SD of asynchronies, onsets in the performance closest to the stimulus were selected. In the analysis reported in the main text, asynchrony values were removed if the interval between performance onset and stimulus onset was longer than 500 ms (i.e., longer than a quarter note). This cleaning step assured that onsets that were missed completely did not affect the analysis. In the analysis presented here, we did not apply this cleaning step to assess the stability of the results.

Results showed that absolute asynchronies were larger in break segments compared to play-along segments (*F*(1,46) = 64.73, *p* < .001, *η*² = .49), and larger in solo compared to duo performances (*F*(1,46) = 5.41, *p* = .025, *η*² = .11; Figure S1A). The two factors did not show a significant interaction (*F*(1,46) = 3.26, *p*= .078, *η*² = .03). The standard deviation of asynchronies showed no significant main effects and no interaction (all *F* < 2.3, all *p* > .13; Figure S1B).

**Reciprocal Influence in Duo Performances without Data Cleaning**

In an additional exploratory analysis following the same steps as described in the main text, we assessed the similarity of asynchronies between “real pairs” and “stranger pairs”. Here, we used all data after only removing double hits without any additional cleaning step.

Results showed a similar pattern as in the cleaned data: Correlations were more positive when the performance was compared to the “real partner” instead of a “stranger” (Figure S2). Although only a trend, the most positive correlations were found for the break segments with the “real partner” (t-test against zero: *t*(15) = 1.95, *p* = .070). All other t-tests against zero were nonsignificant (all *p* > .2).


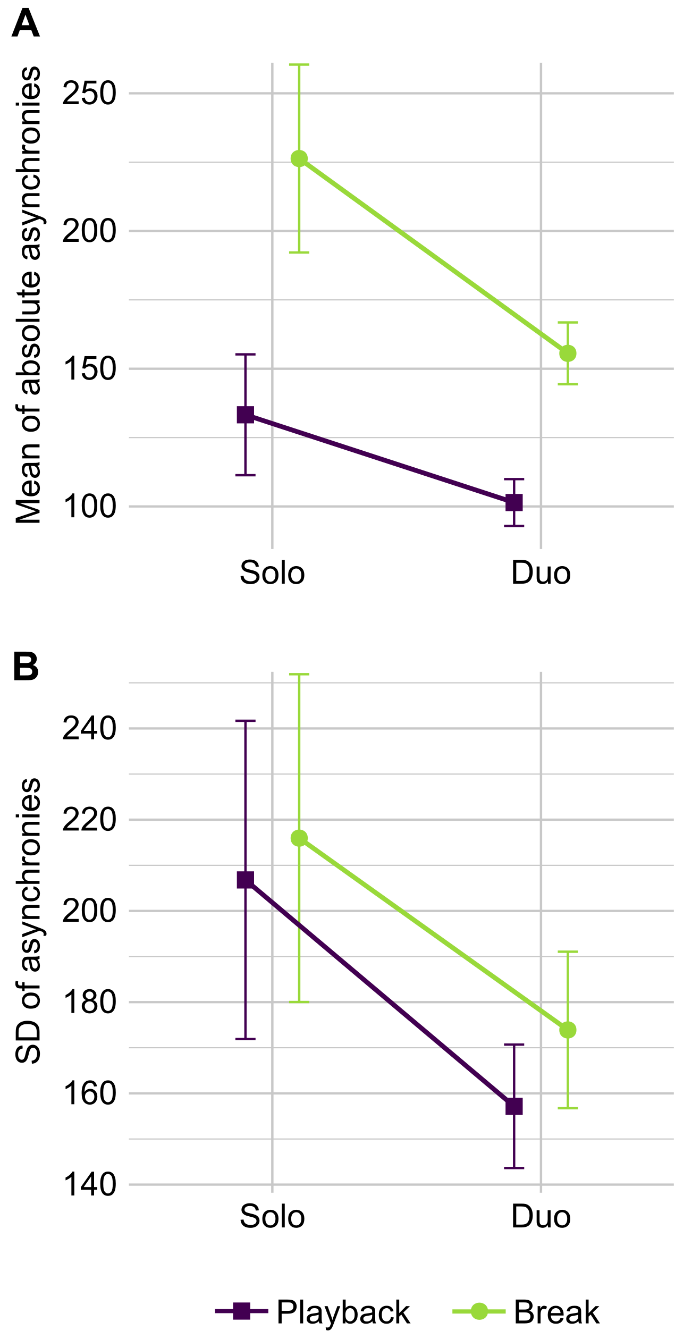


**Figure S1.** Measures of performance quality in duo and solo performances without data cleaning. A) Mean of absolute asynchronies. B) Mean of standard deviations of asynchronies. Error bars represent +/- 1 *SE*.


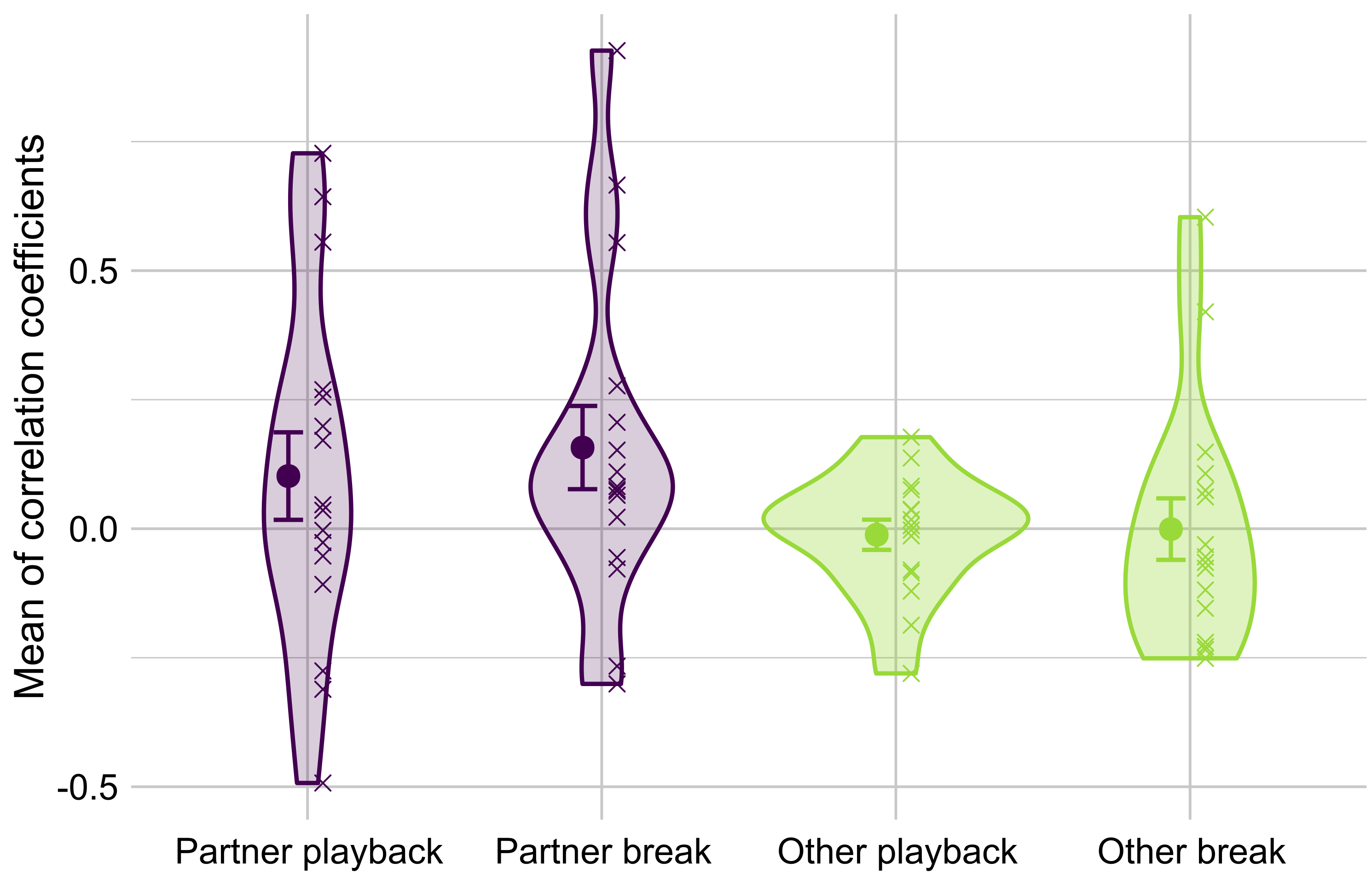


**Figure S2.** Exploratory analysis of the mutual influence of performers. Mean (•) and individual (×) values of Pearson’s correlation coefficients between the mean asynchronies of “real pairs” (ensemble partner) and “stranger pairs” (participants from different duos matched as control condition) during play-along and breaks. Values higher than zero suggest reciprocal influence. The violin plots show the probability density of the data. Error bars represent +/- 1 *SE*.

1. Centre for Systematic Musicology, University of Graz, AT [↑](#footnote-ref-1)
2. Center for Music in the Brain, Aarhus University, DK [↑](#footnote-ref-2)
3. Department of Music, The University of Sheffield, UK [↑](#footnote-ref-3)
